# Evolutionary Conserved Cysteines Function as *cis*-Acting Regulators of *Arabidopsis* PIN-FORMED 2 Distribution

**DOI:** 10.3390/ijms18112274

**Published:** 2017-10-29

**Authors:** Katarzyna Retzer, Jozef Lacek, Roman Skokan, Charo I. del Genio, Stanislav Vosolsobě, Martina Laňková, Kateřina Malínská, Nataliia Konstantinova, Eva Zažímalová, Richard M. Napier, Jan Petrášek, Christian Luschnig

**Affiliations:** 1 Department of Applied Genetics and Cell Biology, University of Natural Resources and Life Sciences, Vienna (BOKU), Muthgasse 18, 1190 Wien, Austria; retzer@ueb.cas.cz (K.R.); nataliia.konstantinova@boku.ac.at (N.K.); 2 Institute of Experimental Botany of the Czech Academy of Sciences, Rozvojová 263, 165 02 Praha 6, Czech Republic; lacek@ueb.cas.cz (J.L.); skokan@ueb.cas.cz (R.S.); lankova@ueb.cas.cz (M.L.); malinska@ueb.cas.cz (K.M.); Zazimalova@ueb.cas.cz (E.Z.); 3 Department of Experimental Plant Biology, Faculty of Science, Charles University, Vinicna 5, 128 44 Prague 2, Czech Republic; stanislav.vosolsobe@natur.cuni.cz; 4 School of Life Sciences, University of Warwick, Gibbet Hill Road, Coventry CV4 7AL, UK; the.paraw@gmail.com (C.I.d.G.); Richard.Napier@warwick.ac.uk (R.M.N.)

**Keywords:** Auxin, PIN proteins, plasma membrane protein sorting, protein mobility, intracellular distribution, root phenotype, *Arabidopsis*, protein modeling, SRRF

## Abstract

Coordination of plant development requires modulation of growth responses that are under control of the phytohormone auxin. PIN-FORMED plasma membrane proteins, involved in intercellular transport of the growth regulator, are key to the transmission of such auxin signals and subject to multilevel surveillance mechanisms, including reversible post-translational modifications. Apart from well-studied PIN protein modifications, namely phosphorylation and ubiquitylation, no further post-translational modifications have been described so far. Here, we focused on root-specific *Arabidopsis* PIN2 and explored functional implications of two evolutionary conserved cysteines, by a combination of *in silico* and molecular approaches. PIN2 sequence alignments and modeling predictions indicated that both cysteines are facing the cytoplasm and therefore would be accessible to redox status-controlled modifications. Notably, mutant *pin2^C−A^* alleles retained functionality, demonstrated by their ability to almost completely rescue defects of a *pin2* null allele, whereas high resolution analysis of pin2^C−A^ localization revealed increased intracellular accumulation, and altered protein distribution within plasma membrane micro-domains. The observed effects of cysteine replacements on root growth and PIN2 localization are consistent with a model in which redox status-dependent cysteine modifications participate in the regulation of PIN2 mobility, thereby fine-tuning polar auxin transport.

## 1. Introduction

Auxin, a versatile plant growth regulator, is involved in a multitude of developmental processes [1,2,3]. This versatility is largely dependent on a very flexible molecular machinery, mediating directional transport of the phytohormone throughout the entire organism [4,5,6]. Plasma membrane localized PIN-FORMED (PIN) proteins, in particular, have been connected to the cellular efflux of the growth regulator, a critical step that defines directionality and rates of polar auxin transport and requires tight regulation [4,7].

Key to the function of PIN proteins at the plasma membrane is a stringent control of their localization, activity and abundance, which has been linked to specific cellular activities. PIN trafficking to and from the plasma membrane is mediated by evolutionary conserved elements of secretory and endocytic sorting machineries, essential for maintenance of and adjustments in PIN distribution. Exocytotic sorting occurs via the TGN (Trans Golgi network), and is dependent on ARF-GEF (ADP Ribosylation Factor–Guanine Nucleotide Exchange Factor) and exocyst complex activities. Plasma membrane-resident PIN proteins are subject to lateral diffusion processes, which is eventually followed by clathrin-dependent endocytic sorting to the TGN. From there, PINs appear to be either rerouted to the plasma membrane, or sorted towards late endosomes en route to the lytic vacuole [4,7].

Some PIN sorting decisions have been linked to reversible, post-translational protein modifications (PTMs), allowing for rapid adjustments in protein function. Phosphorylation of PIN proteins by members of the AGCVIII (cAMP dependent, cGMP dependent, and protein kinase C) protein kinase family has been implicated in the regulation of PIN activity as well as in the control of polar PIN localization at the plasma membrane [8,9,10]. Although it is not yet entirely resolved, how variations in PIN phosphorylation could mediate such responses, structure-function analyses of a number of conserved phosphorylation sites within the PIN central hydrophilic loop revealed their essential function in the regulation of PINs. Specifically, a set of evolutionary conserved serines, found in the N-terminal portion of the central loop domain, has been analyzed extensively and revealed divergent but overlapping roles of distinct phosphosites in polar sorting of PIN proteins and in the activation of PIN-mediated auxin transport across membrane boundaries [9].

Another signal, triggering endocytic sorting of plasma membrane cargo involves reversible covalent attachment of the small protein modifier ubiquitin [11,12]. Recognition of ubiquitylated cargo by distinct adaptor protein complexes triggers a cascade of downstream events, ultimately resulting in cargo delivery to and degradation in lytic vacuoles [13,14]. In case of PIN proteins, ubiquitylation has so far only been demonstrated for *Arabidopsis* PIN2, which is subject to decoration by K63-linked ubiquitin chains [15]. Abolishment of PIN2 ubiquitylation by mutagenesis of several potential ubiquitin attachment sites in the central loop domain caused deficiencies in the protein’s endocytic sorting and functionality, underlining an essential role for ubiquitin-controlled sorting of PIN2 [15].

Next to phosphorylation and ubiquitylation of distinct amino acid side chains, proteinogenic cysteines are subject to a range of different modifications. Cysteines represent the principal target of Reactive Oxygen Species (ROS), with its sulfur atom allowing for several different oxidation states, causing disulfide bond formation, *S*-glutathionylation, or *S*-nitrosylation, to name just a few, with distinct effects on protein fate [16]. Recently, links between auxin and redox signaling [17] were established, as variations in the cellular redox balance, triggered by altered ROS levels, were found to affect auxin-controlled plant morphogenesis [18,19,20]. A key role in controlling the cellular redox status has been attributed to the NADPH-dependent thioredoxin/glutaredoxin (TRX/GRX) and the NADPH-dependent glutathione (GSH) systems, both in the context of preventing oxidative damage as well as in the modulation of redox signaling events [18]. Loss of enzymatic activities, required for regulation of GRX/TRX and/or GSH homeostasis, was found to cause striking developmental aberrations, with combinatorial effects on auxin-controlled development. This is indicated by phenotypes exhibited by an *Arabidopsis ntra ntrb cab* triple mutant, affected in *NTRA* and *NTRB* NADPH-dependent thioredoxin reductase as well as *GSH1* γ-glutamylcysteine synthetase, strikingly resembling mutants defective in polar auxin transport. Moreover, this mutant combination is characterized by a dramatic reduction in *PIN* transcript levels and protein abundance, which could be phenocopied by application of buthionine sulphoximine (BSO), a potent inhibitor of GSH biosynthesis [19,21]. Therefore, it appears plausible that reduced expression of PINs could at least partially account for auxin-related phenotypes described for *ntra ntrb cab*. This hypothesis is supported by another study, demonstrating that *miao*, a leaky *GLUTHATIONE REDUCTASE 2* (*GR2*) loss-of-function allele, markedly interferes with expression of regulators of auxin responses, including *PLETHORA* transcriptional regulators and *PIN* auxin transport proteins [20]. Whether alterations in these mutants’ redox status directly affect distribution and sorting of PIN proteins at a post-transcriptional level remains to be addressed.

Indirect evidence for post-transcriptional regulation of PINs by GSH and/or nitric oxide came from the characterization of an *Arabidopsis* mutant deficient in *S*-nitrosoglutathione reductase (*GSNOR*), catalyzing reduction of GSNO and thereby adjusting levels of *S*-nitrosylated proteins. Specifically, loss of *GSNOR1* in *gsnor1–3* results in severe developmental perturbations, signifying wide-ranging effects of increased protein *S*-nitrosylation [22,23]. Defects involve lowered sensitivity to auxin, indicated by diminished proteolytic turnover of an AXR3/IAA17 reporter protein in response to the hormone as well as reduced lateral root formation, when grown in the presence of synthetic auxin 2,4-D. Moreover, *gsno1–3* seedlings are characterized by a reduction in polar auxin transport, which coincides with diminished levels of PIN auxin transport proteins, similar to observations made for mutants affected in GSH/TRX/GRX homeostasis [23]. However, unlike the situation in *ntra ntrb cab*, PIN protein down-regulation in *gsnor1–3* is not correlated with a corresponding decrease in *PIN* transcript levels [19,23]. In addition, another article reported elevated nitric oxide (NO) levels to cause a reduction of PIN1 protein abundance, without significantly affecting its transcript levels, pointing towards an involvement of protein *S*-nitrosylation in the post-translational control of PIN proteins [24].

Analysis of *Arabidopsis* mutants impaired in GSH and/or NO homeostasis revealed pronounced alterations in PIN protein abundance. However, whether such changes in protein fate arise as a consequence of redox status-induced PTMs of PINs remains unknown. In a pilot approach, we therefore set out and explored the functional significance of highly conserved cysteines found in the PIN protein family, utilizing modeling approaches and site-directed mutagenesis of *Arabidopsis PIN2*. Our findings indicate that, whilst almost dispensable for functionality in root gravitropism, PIN2 cysteines impact on protein distribution, highlighting their potential contribution to the fine-tuning of polar auxin transport.

## 2. Results

### 2.1. PIN Proteins Share Conserved Cysteines that Contribute to Protein Functionality

**Figure 1:**
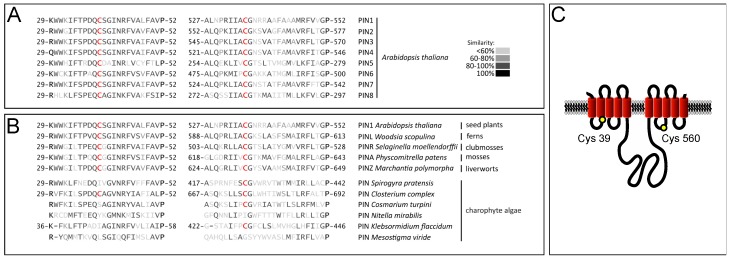
Conservation and predicted localization of conserved PIN cysteines. (**A**) Alignment of *Arabidopsis* PIN protein domains flanking conserved cysteines. Conserved cysteines are displayed in red; (**B**) Alignment of PIN protein domains flanking conserved cysteines, from representative Embryophyta and charophyte green algae. Conserved cysteines are displayed in red. Lack of indicated amino acid positions denotes incomplete sequences; (**C**) 2-D model displaying potential membrane conformation of PIN2. Red barrels represent predicted transmembrane helices, separated by loop domains (black lines). According to these predictions, both cysteines are facing the cytoplasm. The positions of Cys-39 in loop 1 and Cys-560 in loop 7 are indicated (yellow circles).

To address the hypothesis that cysteines do function as *cis*-acting regulators of PIN protein function, we reasoned that such residues should exhibit a high degree of conservation within the PIN family. Multiple sequence alignments performed with *Arabidopsis* PINs revealed a variable number of cysteines encoded by the different members of the gene family. This variability ranged from eight residues found in the *PIN5* ORF (Open Reading Frame) to only two cysteines found in PIN2 (Figure 1A). Notably, these two cysteines are highly conserved in the *Arabidopsis PIN* gene family, which generally appears to be case for PINs encoded by Tracheophyta, including ferns and clubmosses (Figure 1B). Additional alignments performed with predicted canonical PINs from *Physcomitrella patens, Marchantia polymorpha* and additional sequences from Charoyphyta revealed that both cysteines are present in *Marchantia* and *Physcomitrella* PINs, indicative of a high degree of conservation in Embryophyta. In contrast, PIN representatives from charophyte green algae rarely contain both cysteines (Figure 1B).

Taken together, our analysis of PINs found in land plants demonstrates a high degree of evolutionary conservation of a few, distinct cysteines within the protein family.

Among *Arabidopsis* PINs, PIN2 turned out to be unique, as it contains only those two cysteines that are highly conserved in PINs. As the case with any canonical PIN protein, PIN2 hydrophobicity plots indicated the presence of 10 transmembrane helices, which are organized as two blocks of five helices, separated by a central spacer region [25,26,27]. According to these predictions, Cys-39 would localize to the junction between loop 1 and helix 2, whereas Cys-560 would reside between helix 7 and 8. Considering that the PIN central loop region has been predicted to face the interior of the cell [28], then these topology predictions would indicate that both conserved cysteines localize to the cytoplasmic side of the plasma membrane (Figure 1C).

To obtain additional insights into PIN2 topology and accessibility of conserved cysteines, we performed an *in silico* modeling. However, due to the structural heterogeneity of full-length PIN2 (647 aa), we decided that rather than modeling it as a whole entity, it was more feasible to separate it into three constituting parts, and model each one individually, refining then the resulting structures via molecular dynamics (MD) simulations (see Material and Methods for protocol details). Because of the existing predictions of the protein structure [27], we considered the first part of the sequence as going from Met-1 to Arg-155, corresponding to the first cluster of transmembrane helices, the second part going from Gly-156 to Trp-495 and corresponding to the central loop, and the third part spanning from Arg-496 to Leu-647, corresponding to the second cluster of transmembrane helices.

**Figure 2:**
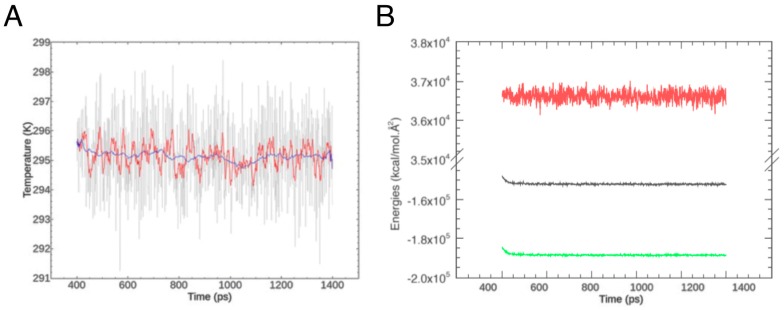
Equilibration of the PIN2 loop. (**A**) Behavior of system temperature over the equilibration period. The full raw data are in grey, while the red and blue lines show window averages over 10 and 100 ps, respectively; (**B**) Potential energy (green), kinetic energy (red) and total energy (black) of the system over the same period.

To generate the initial models for the three parts, we used Modeller [29], producing several hundred structures, from which we selected the three best ones. Then, we refined these via MD simulations using AMBER (http://ambermd.org/) [30]. The procedures we followed are different for the loop and the helix clusters, since the latter are inside the cell membrane, and thus they are surrounded by lipids, rather than by water molecules. Concerning the loop, we solvated it explicitly, and then performed a two-step minimization of the structure: first, we minimized the position of water and ions around the protein fragment by constraining the atoms of the latter to their initial coordinates; then, we removed the constraints and minimized the whole system. Following minimization, we heated up the system with periodic boundary conditions at constant volume, and after the heating phase, we let it equilibrate at constant pressure for 1 ns. The behavior of temperature and energies (Figure 2) shows that equilibration of the loop is indeed reached over the time of these initial MD steps. Finally, we extracted the lowest-energy conformation from the simulation trajectory and minimized it as described above.

**Figure 3:**
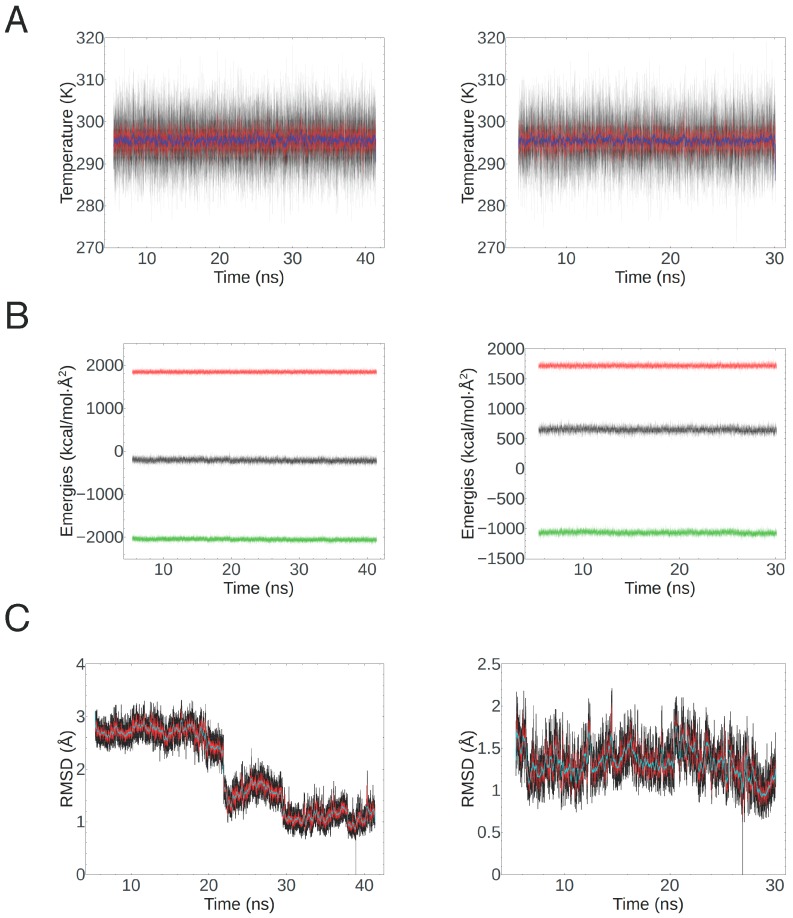
Equilibration and stability of the folded PIN2 helix clusters. (**A**) System temperature of part 1 (**left**) and part 3 (**right**) over the full production run. The raw data are in black, while the red and blue lines show window averages over 10 and 100 ps, respectively; (**B**) Potential energy (green), kinetic energy (red) and total energy (black) of part 1 (**left**) and of part 3 (**right**) over the same period as in (**A**); (**C**) RMSD of the lowest-energy conformation of part 1 (**left**) and part 3 (**right**) with respect to every other frame in the simulation, after alignment (black); the red and light blue lines are window averages over periods of 20 and 200 ps, respectively.

For the helix clusters (parts 1 and 3 of the protein), we employed implicit solvation to approximate the presence of a lipid bilayer as solvent of the protein fragments, which were treated individually and independently. The lipid environment was simulated by imposing a relative permittivity of the implicit solvent equal to 2.2, following the calculation before heating them and performing production runs of part 1 for a total of 41.4 ns and part 3 for a total of 30.1 ns. Temperature and energy plots (Figure 3A,B) show a good stability of the simulation over this time period. Then, we extracted the lowest energy conformations from the trajectories. To check the structural stability of these folded states, we aligned them with each individual frame of the whole simulation, computing a mass-weighted RMSD of the backbones. The results show that the selected conformation for part 1 is stable approximately over the last 20 ns, with an RMSD only occasionally greater than 2 Å and always close to 1 Å for the last 13 ns (Figure 3C). This suggests that no further secondary structure changes are likely to happen. The same conclusion can be drawn about part 3, with an RMSD fluctuating around an average of 1.3 Å for the duration of the production run.

Having obtained MD-refined models for the three regions of PIN2, we stitched them using UCSF Chimera (https://www.cgl.ucsf.edu/chimera/) [31], resulting in a single pdb file of the whole protein on which we performed a final refinement. To start this, we carried out a two-step minimization: first we constrained the positions of the atoms of the transmembrane regions, minimizing the conformation of the loop using the solvent relative permittivity of water; then, we restrained the resulting position of the loop and minimized the transmembrane domains with relative permittivity of the solvent equal to 2.2. To heat up the protein, we decided to constrain the helix clusters and let the loop move freely, since we expect that in the cell environment the rigidity of the loop is considerably smaller than that of the transmembrane domains. Subsequently, we let the loop equilibrate for 1 ns, before constraining it and equilibrating the conformation of the helix clusters for 1 ns. The final, globally equilibrated conformation was then minimized with the same two-step procedure described above.

**Figure 4:**
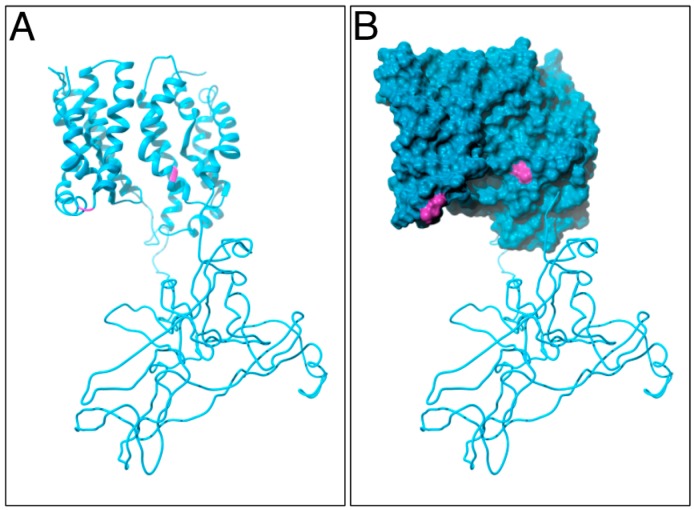
A 3-D model for PIN2: Ribbon diagram (**A**) and surface representation (**B**) of assembled 3-D structural predictions for PIN2. The position of both cysteines is labeled in pink. Rendering was performed within Chimera (https://www.cgl.ucsf.edu/chimera/) [31] using POV-Ray (http://www.povray.org/).

The *in silico* modeling yielded a 3-D structure of the entire, translated PIN2 coding region (Figure 4A,B; Movie S1). Evidently, the calculated protein structure could represent a very useful tool, suitable for addressing PIN function in polar auxin transport. Here, we would like to point out that both cysteines are predicted to be positioned on the surface of modeled PIN2 (Figure 4A,B; Movie S1). If true, then such a protein configuration makes both cysteines accessible for interactions and modifications.

**Figure 5:**
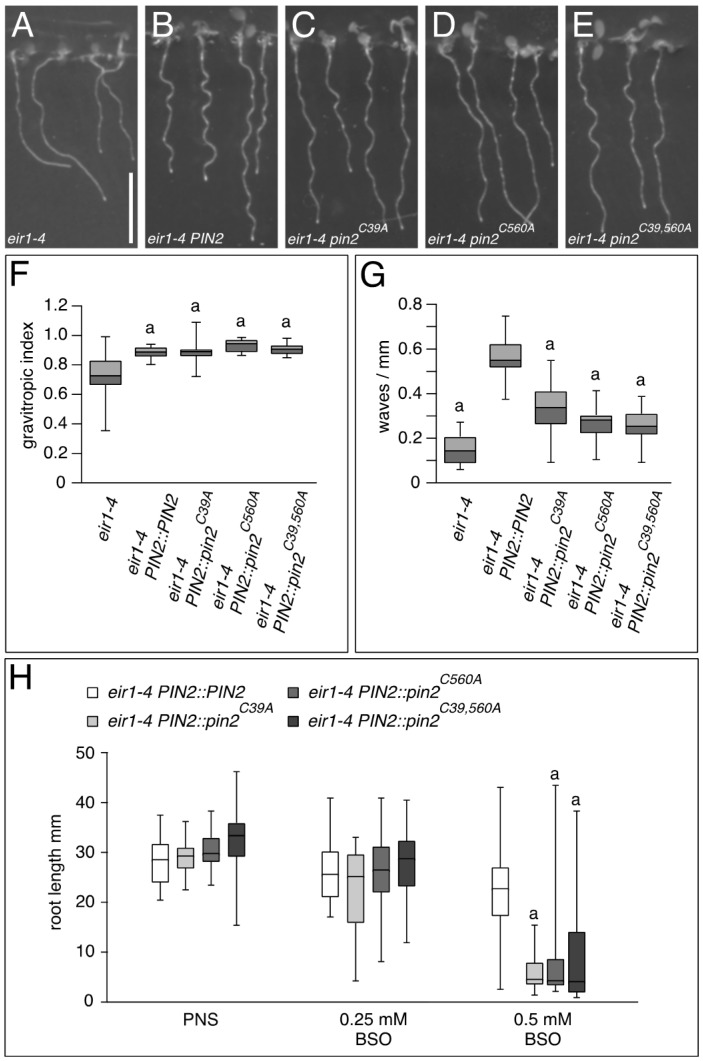
Growth of *pin2^C−A^* alleles. (**A**–**E**) Comparison of: *eir1-4* (**A**); *eir1-4 PIN2::PIN2* (**B**); *eir1-4 PIN2::pin2^C39A^* (**C**); *eir1-4 PIN2::pin2^C560A^* (**D**); and *eir1-4 PIN2::pin2^C39,560A^* seedlings at 6 DAG. Size bar corresponds to 10 mm; (**F**) Box plot displaying the root gravitropic index of *eir1-4*, *eir1-4 PIN2::PIN2*, *eir1-4 PIN2::pin2^C39A^*, *eir1-4 PIN2::pin2^C560A^*, and *eir1-4 PIN2::pin2^C39,560A^*, according to Grabov and colleagues [32] at 6 DAG. In total, 13–27 roots were tested for each genotype. “a” indicates a significant difference to *eir1-4* (*p* < 0.01), determined by One-way ANOVA with post-hoc Tukey HSD test. No significant differences were observed when comparing *eir1-4 PIN2::PIN2* to *eir1-4 PIN2::pin2^C39A^*, *eir1-4 PIN2::pin2^C560A^*, and *eir1-4 PIN2::pin2^C39,560A^*. Whiskers represent the entire range of outliers obtained in the datasets; dark grey and light grey boxes display first and third quartiles, respectively. (**G**) Box plot, displaying the root waviness of *eir1-4, eir1-4 PIN2::PIN2*, *eir1-4 PIN2::pin2^C39A^*, *eir1-4 PIN2::pin2^C560A^*, and *eir1-4 PIN2::pin2^C39,560A^* at 6 DAG, grown on vertically orientated agar (1.5% (*w*/*v*)) nutrient plates. In total, 21–27 roots were tested for each genotype. “a” indicates a significant difference to *eir1-4 PIN2::PIN2* (*p* < 0.01), determined by One-way ANOVA with post-hoc Tukey HSD test. Whiskers represent the entire range of outliers obtained in the datasets; dark grey and light grey boxes display first and third quartiles, respectively; (**H**) Box plot displaying the primary root length of *eir1-4 PIN2::PIN2*, *eir1-4 PIN2::pin2^C39A^*, *eir1-4 PIN2::pin2^C560A^*, and *eir1-4 PIN2::pin2^C39,560A^* at 7 DAG, when germinated in presence of indicated concentrations of buthionine sulphoximine (BSO). In total, 20–32 seedlings were analyzed for each genotype “a” indicates a significant difference to *eir1-4 PIN2::PIN2* (*p* < 0.01), determined by One-way ANOVA with post-hoc Tukey HSD test. Whiskers represent the entire range of outliers obtained in the datasets.

For a functional analysis of PIN2 cysteines, we initiated a site-directed mutagenesis approach, and replaced either Cys-39 or Cys-560 with an alanine residue. In addition, we generated a mutant *pin2^C39,560A^* allele, in which both cysteines were replaced by alanines. These mutant alleles as well as a wild type copy of the *PIN2* ORF were fused to the *Arabidopsis PIN2* promoter and transformed into the root agravitropic *eir1-4* null allele of *PIN2,* in order to study functionality of *pin2^C−A^* alleles [15]. When grown on vertically oriented nutrient agar plates, no striking difference was observed between wild type controls and *eir1-4 PIN2::PIN2* seedlings, demonstrating that the transgene complements loss of endogenous *PIN2* [15]. Similarly, when comparing growth of vertically oriented *eir1-4 PIN2::PIN2* with *eir1-4 PIN2::pin2^C39A^*, *eir1-4 PIN2::pin2^C560A^* and *eir1-4 PIN2::pin2^C39,560A^* seedlings, we did not observe any prominent differences in gravitropic root growth, which was indistinguishable from growth of wild type Col-0 seedlings, indicating that *pin2^C−A^* alleles have retained functionality, sufficient for rescuing major *eir1-4* growth deficiencies (Figure 5A–F). Closer examination of root growth, however, revealed a reduction in root waviness for all three different *pin2^C−A^* alleles, when grown on the surface of vertically positioned agar medium (Figure 5A–E,G). Thus, whilst mutagenesis of cysteines does not interfere with overall PIN2 functionality in root gravitropism, these residues nonetheless appear critical for fine-tuning of directional root growth.

Pharmacological interference with GSH homeostasis by treatment of *Arabidopsis* seedlings with BSO resulted in pronounced defects in root development, which coincided with reduced expression of *PIN* genes [19,21]. In related experiments, we tested growth of *pin2^C−A^* alleles in response to BSO, which demonstrated increased root growth inhibition of *eir1-4 PIN2::pin2^C39A^*, *eir1-4 PIN2::pin2^C56^^0^^A^* and *eir1-4 PIN2::pin2^C39,56^^0^^A^*, when germinated in presence of the drug (Figure 5H). Notably, this apparent hypersensitivity to BSO treatment, was visible, even when testing *pin2^C39,56^^0^^A^* completely lacking cysteines. This observation argues for additive effects of BSO-induced downregulation of GSH biosynthesis and Cys-to-Ala substitutions introduced into *pin2^C−A^* alleles. Whilst the nature of this interaction remains to be determined, it supports a role for the conserved cysteines in the regulation of PIN2 function.

Taken together, mutational analysis of PIN2 cysteines revealed their requirement for full functionality of the auxin transport protein. Nevertheless, expression of *pin2^C−A^* alleles is sufficient to rescue principal *eir1-4* growth deficiencies, indicating that these point mutations do not generally abolish PIN2 activity. Subtle growth deficiencies associated with *pin2^C−A^* alleles, would rather argue for a role of PIN2 redox control in the fine-tuning of adjustments in root growth.

### 2.2. Conserved Cysteines Are Determinants of PIN2 Intracellular Distribution

**Figure 6:**
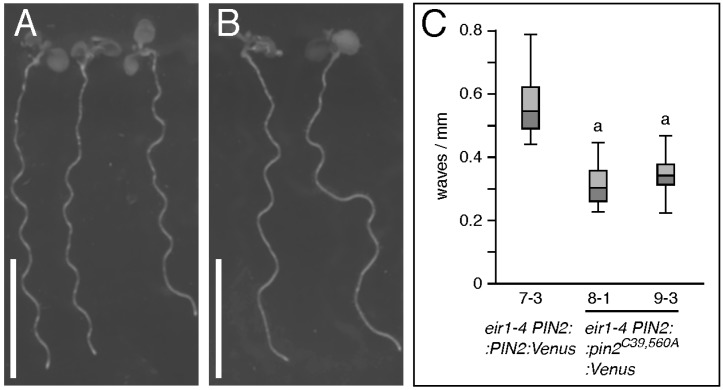
Analysis of *PIN2::pin2^C39,560A^:Venus* lines. (**A**,**B**) Comparison of: *eir1-4 PIN2::PIN2:Venus* (**A**) and *eir1-4 PIN2::pin2^C39,560A^:Venus* (**B**) seedlings at 6 DAG grown on vertically oriented nutrient plates. Size bars correspond to 10 mm; (**C**) Root waviness of *eir1-4 PIN2::PIN2:Venus* and two independent *eir1-4 PIN2::pin2^C39,560A^Venus* lines at 6 DAG, grown on vertically orientated agar (1.5% (*w*/*v*)) nutrient plates. In total, 25–28 seedlings were tested for each genotype. “a” indicates a significant difference to *eir1-4 PIN2::PIN2:Venus* (*p* < 0.01), determined by One-way ANOVA with post-hoc Tukey HSD test. Whiskers represent the entire range of outliers obtained in the datasets; dark grey and light grey boxes display first and third quartiles, respectively.

To test for possible consequences of *pin2^C−A^* mutations on intracellular distribution and sorting of PIN2, we generated Venus-tagged translational fusion constructs, which were then expressed in *eir1-4*. The resulting *eir1-4 PIN2::pin2^C39,560A^:Venus* lines exhibited phenotypes similar to those of lines expressing untagged *pin2^C−A^* alleles (Figure 6A–C), and were subject to further expression analysis.

**Figure 7:**
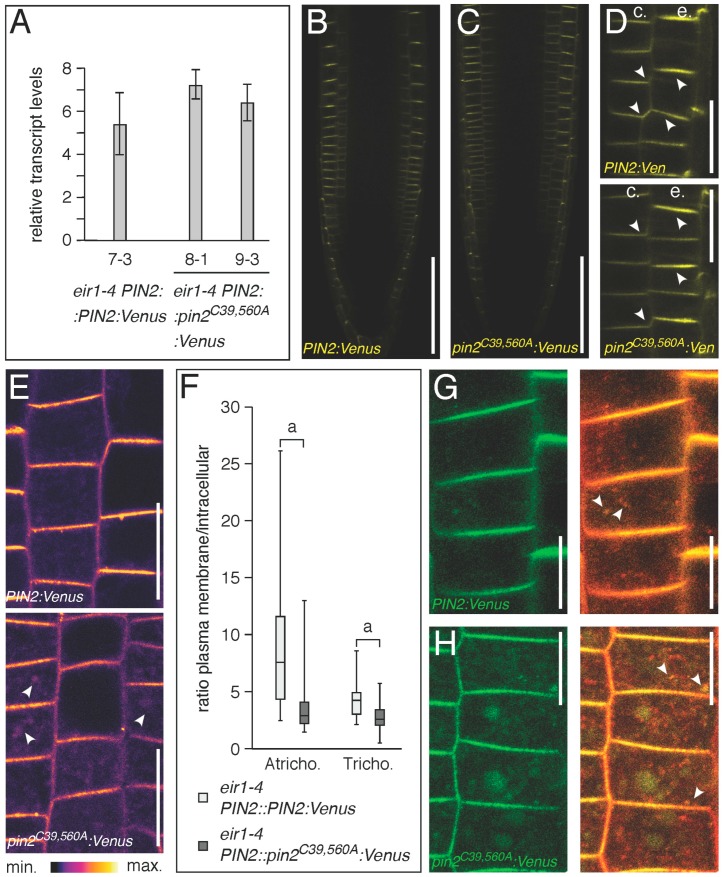
Expression pattern and localization of *PIN2::pin2^C39,560A^:Venus* reporter lines. (**A**) Relative transcript levels of *PIN2::PIN2:Venus* and two *PIN2::pin2^C39,560A^Venus* lines at 6 DAG. Two biological repetitions have been used for each sample, with transcripts normalized to expression of *EF1a* (At1g07940). Bars indicate standard deviations. One-way ANOVA with post-hoc Tukey HSD test demonstrated no significant differences in transcript levels (*PIN2::PIN2:Venus* vs. *PIN2::pin2^C39,560A^Venus* 8-1, *p* = 0.1506725; *PIN2::PIN2:Venus* vs. *PIN2::pin2^C39,560A^Venus* 9-3, *p* = 0.5558223); (**B**,**C**) Expression pattern (yellow coloration) in: *eir1-4 PIN2::PIN2:Venus* (**B**) and *eir1-4 PIN2::pin2^C39,560A^Venus* (**C**) primary root meristems at 6 DAG is restricted to lateral root cap, epidermis and cortex cells; (**D**) Reporter signal localization in epidermis (“e”) and cortical (“c”) in *eir1-4 PIN2::PIN2:Venus* and *eir1-4 PIN2::pin2^C39,560A^Venus* primary root meristem cells at 6 DAG. Arrowheads indicate polar localization of the reporter signals; (**E**) Comparison of signal distribution in *eir1-4 PIN2::PIN2:Venus* and *eir1-4 PIN2::pin2^C39,560A^Venus* primary root meristem epidermis cells at 6 DAG; white arrowheads indicate intracellular reporter protein signals; (**F**) Signal quantification in *eir1-4 PIN2::PIN2:Venus* and *eir1-4 PIN2::pin2^C39,560A^Venus* primary root meristem epidermis cells at 6 DAG. The ratio of reporter signal intensities at the plasma membrane compared to intracellular signals was determined in 36–57 trichoblast (“Tricho.”) and atrichoblast (“Atricho.”) cells for each sample. Two-tailed *t*-test analysis of resulting values demonstrated significant differences (*p* < 0.001, “a”); (**G**,**H**) Staining of *eir1-4 PIN2::PIN2:Venus* and *eir1-4 PIN2::pin2^C39,560A^Venus* (green) with FM4-64 for 30 min in the dark, followed by visualization at the CLSM. White arrowheads indicate co-staining (yellow) in endocytosed compartments. Size bars: **B**–**D** = 50 μm; **E** = 20 μm; **G**,**H** = 10 μm.

*Eir1-4 PIN2::pin2^C39,560A^:Venus* exhibited an expression pattern and polarly localized signals at the plasma membrane, similar to wild type PIN2:Venus (Figure 7A–D). However, when comparing subcellular distribution of PIN2:Venus and pin2^C39,560A^:Venus protein in more detail, we observed an increase of intracellular signals in epidermal cells of *eir1-4 PIN2::pin2^C39,560A^:Venus* seedlings (Figure 7E). This increase was apparent in trichoblast and atrichoblast cells of primary root meristems, indicative of modifications in intracellular distribution/sorting of the mutant PIN2 reporter protein (Figure 7F). Furthermore, when performing co-staining experiments with the endocytosed styryl dye FM4-64, we detected a prominent overlap in signal distribution, demonstrating that mutant pin2^C39,560A^:Venus has a strong tendency to enter the endocytic sorting pathway, which might explain the increased intracellular accumulation of pin2^C39,560A^:Venus signals (Figure 7G,H).

We reasoned that the apparent differences in intracellular distribution of PIN2:Venus and pin2^C39,560A^:Venus signals highlight a requirement for the conserved cysteines in PIN protein traffic.

**Figure 8:**
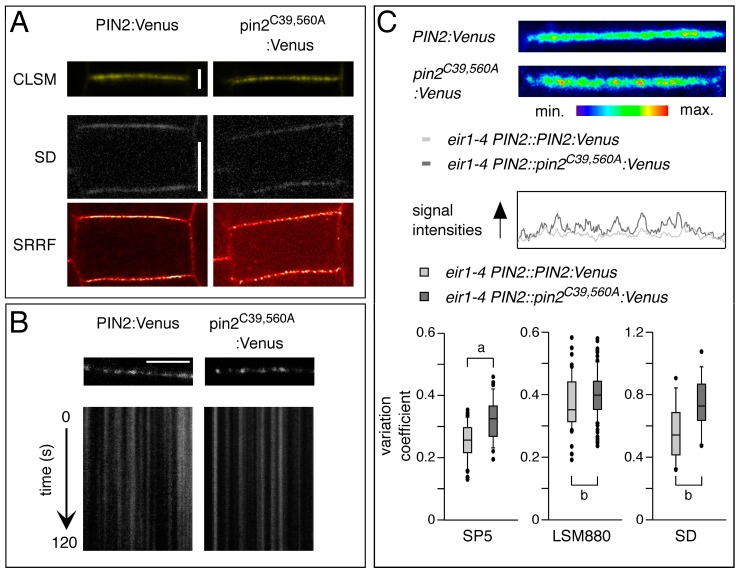
Distribution and mobility of PIN2-Venus reporter proteins. (**A**) Upper row: Comparison of PIN2:Venus (**left**) and pin2^C39,560A^:Venus (**right**) signals at the plasma membrane of root epidermis cells, viewed by conventional CLSM imaging. Middle row: A single frame generated by fast SD scanning (50 ms exposition), displaying PIN2:Venus (**left**) and pin2^C39,560A^:Venus (**right**) signals at the plasma membrane of root epidermis cells. Bottom row: SRRF algorithm applied to 100 frames generated by SD on the same sections as above. Scale bars 2 µm (CLSM) and 5 µm (SD); (**B**) Kymographs representing the fluorescence of PIN2:Venus (left) and pin2^C39,560A^:Venus (right) signals over time. Scale bar 5 µm; (**C**) Top panel: Comparison of signal distribution in root epidermis plasma membrane domains of PIN2:Venus and pin2^C39,560A^:Venus. Bottom panels: Box plots displaying PIN2:Venus and pin2^C39,560A^:Venus signal intensity profiles at the plasma membrane calculated from CLSM (Leica SP5, Zeiss LSM880) and SD images. Ninety membranes from images generated by Leica SP5, 150 from Zeiss LSM880 and 40 membranes from SD images were used for these analyses. Two-tailed *t*-test analysis of resulting values demonstrated significant differences (*p* < 0.001, “a”; *p* < 0.05, “b”).

Models for PIN2 sorting in root epidermis cells predicted super-polar exocytotic sorting to the central section of the apical plasma membrane domain. A fraction of plasma membrane-targeted PIN2 was suggested to be subject to lateral diffusion, followed by clathrin-dependent endocytosis, initiated at the outermost edges of the apical domain. In contrast, another fraction of exocytosed PIN2 was described to accumulate in plasma membrane-associated clusters, exhibiting only limited mobility, when compared to the kinetics of other plasma membrane proteins [33,34]. This led to models in which the interplay between mobile vs. immobile PIN2 fractions at the plasma membrane could determine abundance, distribution and hence PIN2 activity in cellular auxin efflux [33]. We analyzed PIN2:Venus and pin2^C39,560A^:Venus reporter protein signals at the plasma membrane of root epidermis cells by using conventional confocal microscopy. This analysis revealed discontinuities in signal distribution along the plasma membrane, which might reflect the occurrence of PIN2 signal aggregates (Figure 8A). Attempts to visualize these structures at higher resolution by super-resolution STED microscopy were not successful, due to very prominent signal bleaching, specifically of pin2^C39,560A^:Venus, and we therefore utilized an alternative high resolution approach.

The Super-Resolution Radial Fluctuations (SRRF) algorithm has been introduced recently, to allow for generation of high resolution images, with illumination intensity requirements orders of magnitude lower, than used by other super-resolution methods [35]. We made use of this approach, which allowed us a comparison of signal distribution and signal intensities in *eir1-4 PIN2::PIN2:Venus* and *eir1-4 PIN2::pin^C39,560A^:Venus* root meristem cells, derived from frame sets generated by fast scanning with low laser power Spinning Disc (SD) confocal microscopy. This sub-diffraction analysis revealed distinct structures in the sub-nanometer size range in *eir1-4 PIN2::PIN2:Venus* and *eir1-4 PIN2::pin^C39,560A^:Venus* root meristem cells (Figure 8A), likely reflecting PIN2 clusters that have been described earlier [33]. We then employed SD confocal microscopy and kymograph analysis to determine signal distribution over time. These experiments indicated very limited cluster movement when analyzing either wild type PIN2:Venus or pin2^C39,560A^:Venus signals (Figure 8B). From that, we concluded that formation of static PIN2 clusters at the plasma membrane is not categorically obstructed by mutagenesis of PIN2 cysteines.

To assess the distribution of PIN2:Venus signal at the plasma membrane we decided to evaluate local fluctuations of fluorescence intensity in membranes of individual cells. The resulting values were used for calculating the coefficient of variation of signal intensity profiles along plasma membranes in PIN2:Venus and pin2^C39,560A^:Venus lines. Notably, evaluation of these profiles revealed a prominent increase in the coefficient of variation in pin2^C39,560A^:Venus signal intensities, when compared to the wild type protein. Higher signal variabilities point towards a less homogenous distribution of the mutant reporter protein at the plasma membrane, indicative of local alterations in protein distribution (Figure 8C). Thus, apart from the increased intracellular accumulation of pin2^C39,560A^:Venus, Cys-to-Ala substitutions seemingly affect the pattern of PIN2 distribution within plasma membrane micro-domains, hinting at an overall altered mobility of the mutant protein.

## 3. Discussion

Timely and coordinated responses to environmental parameters are important for the survival of sessile organisms, well exemplified by the ability of higher plants to adapt to a wide range of growth conditions. At the molecular level, adaptations to variable conditions involve a diversity of PTMs, influencing distribution and mobility, steady state levels, conformation as well as activity of proteins. Specifically, modifications of cysteines play a decisive role in such post-translational control of proteins, and were found to be of general importance for all life on earth [16]. In this study, we tested the role of two conserved cysteines identified in the PIN family of auxin transport proteins, revealing a role in protein mobility and localization in plasma membrane micro-domains. Such altered PIN2 behavior could be attributed to modifications in protein topology, caused by the point mutations introduced, and would be consistent with a role of redox status-controlled cysteine modifications in the regulation of PIN2 mobility.

At present, crystal structure and overall configuration of PIN proteins are not known, which makes it difficult to draw conclusions solely based on the outcome of site-directed mutagenesis experiments, as described in this study. Conversely, aquaporin-type water channel proteins, represent extremely well characterized plasma membrane proteins, and have been subject to extensive analyses [36]. Notably, mutagenesis of a conserved cysteine found in plant PLASMA MEMBRANE INTRINSIC PROTEIN-(PIP)-type aquaporins had effects similar to those that we observed for mutant pin2^C−A^. Specifically, a PIP Cys-to-Ser substitution did not significantly interfere with protein function, as it had no striking effects on protein sorting and protein activity, upon heterologous expression in oocytes. Moreover, formation of the biologically active, tetrameric PIP configuration remained unaffected by this mutation [37], questioning a contribution of the conserved cysteine to PIP maturation and oligomerization. Consistent with these findings, another study identified several transmembrane helix-localized residues as critical for intra- and inter-proteinogenic helix interactions, which turned out to be essential for PIP sorting to the plasma membrane and for formation of functional PIP protein tetramers [38]. Based on these observations, it appears that disulfide bond formation plays only subordinate roles in establishing functional PIP protein complexes.

By analogy to PIPs, our analysis of mutant pin2^C−A^ suggests that disulfide bond formation is not a principal prerequisite for activity of the auxin transport protein. This is indicated by the apparently polar localization of pin2^C39,560A^:Venus, together with rather subtle root growth defects, associated with expression of *pin2^C−A^* alleles in a *pin2* null background. This contradicts a model, in which redox status-induced PTMs are quintessential for the regulation of PIN2 polar targeting. Rather, we conclude that PIN2 cysteine residues and potential redox signaling-controlled modifications contribute to association to specific plasma membrane domains, thereby influencing PIN2 sorting processes.

A report by Kleine–Vehn and colleagues [33] provided evidence for the existence of distinct PIN protein pools that can be found at plasma membrane domains. The majority of membrane-associated PIN2 appears to form protein aggregates, visible as irregularly shaped signal clusters with a limited tendency to move within the plasma membrane. In addition, another protein fraction was suggested to show higher mobility, which is primarily based on observations, demonstrating clathrin-dependent PIN2 endocytosis at distal portions of the apical plasma membrane domain of root epidermis cells. The biological significance of such distinct PIN2 fractions and mechanisms that would control the equilibrium between such fractions, however, are not known [33].

PIN2 super-resolution SRRF analyses presented in this study, provides additional evidence for the existence of distinct PIN2 pools. This is supported by signal quantification, based on SRRF-generated images, together with SD analysis, demonstrating aggregations of immobile PIN2 signals for PIN2 wild type and pin2^C39,560A^ reporter lines. However, when determining intensity profiles by calculation of reporter protein signal scattering, we observed a prominent increase in the local variations of pin2^C39,560A^ signal intensities. These variabilities reflect adjustments in protein localization within plasma membrane micro-domains, potentially arising from an altered intramembranous mobility caused by Cys-to-Ala substitutions introduced into PIN2. We cannot categorically exclude a scenario, in which an altered protein conformation caused by the point mutations introduced, is solely responsible for the altered characteristics of pin2^C39,560A^. Due to the differences in their side chains, replacement of cysteines by alanines might cause subtle changes in the overall structure of protein domains. This however, might also be true for serine, frequently used for mutational analysis of cysteine residues, and characterized by a hydroxyl group instead of a sulfhydryl group in its side chain. These side chains enable hydrogen bond formation between serines, when positioned in an adequate distance, thereby influencing protein conformation. A detailed biochemical analysis of PIN2 protein conformation and of potential redox status-controlled effects on PIN2 is required, to further address this issue.

Indirect support for redox status-dependent PIN protein modifications comes from experiments, demonstrating that variations in NO levels and protein *S*-nitrosylation exert strong effects on PIN abundance at the plasma membrane [22,23]. As a result, redox status-dependent variations in PIN distribution would modulate auxin flow, via controlling protein mobility in the plasma membrane. PIN2 thus could be subject to sorting control mechanisms, similar to those of *Arabidopsis* Salicylic Acid (SA) receptor *NONEXPRESSER OF PR GENES 1* (*NPR1*), which undergoes redox status-dependent protein redistribution in response to pathogens. NPR1, a regulator of plant systemic acquired resistance (SAR), resides in the cytoplasm and in the nucleus, where it functions as transcriptional co-regulator of *PR* genes [39]. Cytoplasmic sequestration depends on intermolecular disulfide bond formation between NPR1 monomers, which is promoted by *S*-nitrosylation of NPR1 [40]. Conversely, SA-induced reduction of NPR1 oligomers, facilitated by thioredoxins, results in nuclear accumulation of monomeric NPR1, where it modulates expression of defense-related genes [39,40,41]. This elegant mechanism integrates stimulus-dependent variations in redox signaling and plant defense responses via control of NPR1 conformation, and a related scenario could be envisioned for PIN2. By analogy to NPR1, redox status-induced structural changes of PIN2 could be induced by reversible cysteine modifications, which might influence conformation of the protein. This in turn, could transiently affect PIN2 mobility, followed by altered distribution in plasma membrane micro-domains and variations in endocytic protein sorting.

At this moment, we are still lacking conclusive experimental evidence for redox status-dependent PTMs directly controlling mobility and sorting of PIN2. Specifically, whilst some reports demonstrated dramatic effects of NO-signaling on post-transcriptional regulation of PIN protein abundance, it remains to be determined, whether or not PINs represent substrates for associated protein modifications [23,24]. This is also the case for our understanding of mechanisms, by which redox-controlled PTMs could affect mobility of proteins at the plasma membrane. A recent study suggested cross-talk between plant plasma membrane proteins and the cell wall as a determinant of protein mobility, and such cross-talk appears essential for correct sorting and localization of PIN proteins as well [34,42]. Adjustments in redox signaling could modify such hypothetical PIN2–cell wall interactions in a quantitative manner, thereby influencing PIN2 mobility and function at the plasma membrane.

Regardless of the hypothetical role of PIN2 cysteines in redox signaling, subtle phenotypes of *pin2^C−A^* alleles suggest that adjustments in the PIN2 redox status exert only limited effects on polar auxin transport and root growth. These mild developmental modifications are in striking contrast to the severe auxin-related defects, described for mutants defective in the control of GSH and/or NO homeostasis [19,20,23]. Hence, it appears that transmission of redox signals that decide about *PIN* expression and activity depends on a combination of events [17,19,20,23,43]. Deciphering the interplay between such distinct redox status-controlled processes and how they might jointly shape auxin distribution and signaling events in the orchestration of plant development, remains a challenge for future research.

## 4. Materials and Methods

### 4.1. Plant Lines, Growth Conditions and Vector Construction

Plants were grown on ½× Murashige Skoog medium, or on PNS plant nutrient agar plates (5 mM KNO_3_, 2 mM MgSO_4_, 2 mM Ca(NO_3_)_2_, 250 mM KPO_4_, 70 µM H_3_BO_3_, 14 µM MnCl_2_, 500 nM CuSO_4_, 1 µM ZnSO_4_, 200 nM Na_2_MoO_4_, 10 µM NaCl, 10 nM CoCl_2_, 50 µM FeSO_4_; pH adjusted to 5.7; supplemented with 1% (*w*/*v*) agar and 1% (*w*/*v*) sucrose; in a 16 h light/8 h dark regime at 22 °C). *PIN2::PIN2*, *PIN2::PIN2:Venus* and *eir1-4* have been described elsewhere [15,44]. For site-directed mutagenesis, we generated primers 5′-GGGATATTCACACCGGACCAAGCTTCCGGTATAAACCGGTTGC-3′ and 5′-CGAACCGGTTTATACCGGAAGCTTGGTCCGGTGTGAATATCC-3′ for mutagenesis of C39. As a template for PCR we made use of *PIN2::PIN2* and *PIN2::PIN2:Venus* binary vector constructs, as described previously. *Resulting pin2^C39A^* candidate clones were confirmed by sequencing and subsequently subject to another round of site-directed mutagenesis, using primers 5′-AACCAAAGATTATTGCGGCCGGAAAATCAGTAGCAGGG-3′ and 5′-CCCTGCTACTGATTTTCCGGCCGCAATAATCTTTGGTT-3′ for replacement of C560. Resulting *pin2^C39,560A^* candidate clones were confirmed by sequencing. For generation of *pin2^C560A^*, PIN2::PIN2 and PIN2::PIN2:Venus binary vectors were used as template DNA. Flowering *Arabidopsis* plants were transformed by the floral dip method [45], using *Agrobacterium tumefaciens* strain GV3101/pMP90 [46]. Resulting T2 lines were confirmed for single transgene insertion sites and propagated to homozygosity for further analyses.

### 4.2. Microscopy

Spinning disk confocal microscopy was performed using spinning disc microscope Eclipse Ti-E (Nikon, Tokyo, Japan) with CSU-X1 SD unit (Yokogawa, Tokyo, Japan), 100× Plan-Apochromat objective (NA = 1.45 Oil) and a dual camera system. Time series for SRRF analysis were acquired with an EMCCD camera iXon3 897 (Andor Technology, Belfast, Northern Ireland) obtaining images with no averaging at 32 fps with a pixel size of 110 nm. Kymograph imaging was performed with a sCMOS camera Zyla (Andor Technology). Fluorescence signals were excited with a diode laser (488 nm; Agilent, Santa Clara, CA, USA) and fluorescence emission recorded using Semrock Brightline single-pass filters (Semrock, Rochester, NY, USA) for GFP or YFP. For kymograph analysis, a 120 s time series was taken for each image.

CLSM images were generated using Leica SP5 (Leica Microsystems, Wetzlar, Germany) and Zeiss LSM880 (Carl Zeiss, Jena, Germany) microscopes. For imaging, we used the following excitation conditions: 514 nm (Venus), 561 nm (FM4-64). For endocytic sorting studies, 5–6 day old seedlings were transferred from horizontally oriented nutrient plates into 6-well plates with liquid medium and incubated in presence of FM4-64 (Invitrogen, Waltham, MA, USA; working concentration 2 µM) for 30 min before CLSM visualization.

### 4.3. Homology Modeling and Molecular Dynamics

To find appropriate templates to model the three sequence parts, we submitted them to PSIPRED for analysis via GenTHREADER [47,48,49,50], which identified the sodium bile acid symporter from *Y. frederiksenii* (PDB code 4N7W) as a viable template for parts 1 and 3, and the capsid of the B19 parvovirus (PDB code 1S58) as a homologous structure for part 2. We then employed Modeller, a program that implements several methods to determine the structure of proteins via comparative modeling. These were obtained, using the “slow” library scheduler and the “very slow” annealing refinement, as these options provide the highest level of modeling accuracy. Each base model of part 2 was then refined via loop optimization 16 independent times, yielding a total of 1024 models for the central loop. Loop optimizations were also performed with the “very slow” annealing, and repeated twice for each of the 16 independent refinements. To choose the best models for each part, we considered the respective values of the Modeller objective function, the high-resolution discrete optimized protein energy (d atomic potential).

To prepare the input for the molecular dynamics simulations, we explicitly solvated part 2 of the protein in an octahedral box with a minimum allowed distance between solute atoms and box edges of 8 Å and the addition of 5 Cl^−^ counter-ions to neutralize the electric charge. To prepare the input for the molecular dynamics simulations, we explicitly solvated part 2 of the protein in an octahedral box with a minimum allowed distance between solute atoms and box edges of 8 Å and the addition of 5 Cl^−^ counter-ions to neutralize the electric charge. The model we used for implicit solvation of the transmembrane helix clusters (parts 1 and 3), as well as for the final, assembled protein, is the Generalized Born with volume corrections of Ref. [51], with further optimizations for H, C, N, O and S atoms in proteins as described in Ref. [52]. The topology parameters for the loop were prepared using the ff14SB force field for the protein fragment [53] and the TIP3P model for the water molecules [54]. For parts 1 and 3, and for the full protein, we used instead the ff14SBonlysc force field, which includes the backbone parameters of the ff99SB force field [55] with the side-chain parameters of the ff14SB [53], and which is known to yield the best results with the implicit solvation model we chose.

All minimization steps were automatically stopped when the RMS of the Cartesian components of the energy gradient became smaller than 0.05 kcal/(mol·Å^2^). Whenever applied, positional constraints on the atoms were obtained by applying a harmonic potential, with force constant of 500 kcal/(mol·Å^2^) for part 2, and 20 kcal/(mol·Å^2^) for the full protein.

Heating was always carried out to a temperature of 295.15 K over a time of 400 ps with a time-step of 2 fs, constraining the bond length of the protein fragment with SHAKE [56] and that of water molecules, whenever present, with SETTLE [57]. The force constant for the harmonic potential restraints during heating of the full assembled protein was 20 kcal/(mol·Å^2^).

During equilibration and production runs, temperature control was achieved via Langevin dynamics. The collision frequency was 2 ps^−1^ for the simulations of the three individual pieces, and 5 ps^−1^ for the final full protein, where we wanted to maintain a tighter thermal coupling.

For the simulations of part 2, long-range electrostatic interactions were evaluated via a particle-mesh Ewald procedure with a cutoff of 8 Å. The implicit solvent simulations were always carried out with an effectively infinite cutoff (larger than system size) for the non-bonded pair truncation and for the maximum atom distance to consider in the effective Born radii calculation, with the exception of the heating and loop equilibration steps for the assembled proteins, where we imposed a cutoff of 16 Å. In addition, during the implicit solvent simulations, forces dependent on effective radii derivatives and interactions over more than 8 Å were computed every two integration steps.

### 4.4. Data Acquisition and Processing

For root elongation assays, seedlings of each genotype were germinated on PNS in presence of the indicated drugs. After incubation on vertically positioned nutrient plates, seedlings were scanned and root length was determined, using ImageJ/Fiji software [58]. For root gravitropism and root waving assays, seedlings of each genotype were germinated on vertically oriented nutrient plates. Seedlings were scanned and resulting images used for determination of the gravity index [32] and root waves per root length. For assessment of reporter protein distribution, we determined relative grey values at the plasma membrane and in endocytic/vacuolar compartments, by using Fiji/ImageJ software [58].

Images acquired with Leica SP5, Zeiss LSM880 and spinning disc microscope were used to evaluate protein accumulation in membrane micro-domains. For the quantification of CLSM fluorescence at the plasma membrane, intensity profiles along individual membranes were generated with Zen Blue software (Zeiss). For each membrane, the coefficient of variation was calculated as the ratio of standard deviation to the mean florescence intensity, taking into account all fluorescence intensities of individual pixels. These coefficients of variation were used as measures for local fluctuations of fluorescence intensity in membranes of individual cells. Average values were depicted as box plots, their statistical significance was calculated using Two-tailed *t*-test or Mann-Whitney Rank Sum test in Sigma Plot (Systat, Chicago, IL, USA).

For SRRF analysis, the NanoJ-SRRF plugin for Fiji software was used [30]. Image stack of 100 SD image frames (frequency 32 fps) was grabbed with NIS elements 3.1 software (Nikon, Tokyo, Japan) in 14 bit color depth, image resolution 512 × 512 and pixel size 110 nm. Whole image stacks were converted to NanoJ file format and processed with SRRF analysis function with default values (ring radius 0.5, radiality magnification 5 and 6 axes in ring). No drift correction was needed, because there was no detectable growth and/or shift of cells during fast scanning. Resulting SRRF images with resolution 2560 × 2560 were obtained.

### 4.5. PIN Sequences and Alignments

PIN sequences from streptophyte algae were obtained from the NCBI SRA databases [59]. The sequence from *Klebsormidium flaccidum* was used as a query for tblastn search [60]. Short reads were assembled with CAP3 [61] and resulting contigs were subjected to further blastn searches against the respective SRA database to achieve additional sequence coverage. The obtained algal sequences were added to an alignment of representative land plant *PIN* sequences published previously [27] by means of the realigning MAFFT algorithm—add method with automatic parameter adjustment [62]. The degree of amino acid conservation reflected by the black–white color gradient was determined with the Geneious program (Blosum62 matrix; threshold set to 1).

### 4.6. Expression Analysis

RNA was isolated from 6-days old *Arabidopsis* seedlings using RNeasy Plant Mini kit (Qiagen, Hilden, Germany), treated with DNase I (Ambion, Waltham, MA, USA) and reverse transcribed using oligo-dT primers and M-MLV Reverse Transcriptase (Promega, Fitchburg, WI, USA). First strand cDNA was diluted 20× and qPCR was performed using the GoTaq^®^ qPCR Master Mix (Promega) at 58 °C on a LightCycler 480 (Roche, Basel, Switzerland). The relative ratio of target gene expression was calculated using the equation: ratio = (eff_ref_^CPr^)/(eff_target_^CPt^), with eff_ref_ representing PCR efficiency of the reference gene and eff_target_ representing PCR efficiency of the target gene. CPr and CPt represent crossing points of reference and target gene, respectively [63].

Primers pairs used for *PIN2* reporters were 5′-ATTGCTTAGGGCGATGTACG-3′ 5′-TAATTGAACCAGCCGTCTCC-3′ as well as 5′-GGGGTGGTGCCCATCCTGGTCG-3′ 5′-CCTCGGCGCGGGTCTTGTAG-3′. For amplification of the reference gene *EF1a* (At1g07940) 5′-TGAGCACGCTCTTCTTGCTTTCA-3′ and 5′-GGTGGTGGCATCCATCTTGTTACA-3′ were used.

## Supplementary Materials

Supplementary materials can be found at http://www.mdpi.com/1422-0067/18/11/2274/s1.
