# Supplementary material for: Evolutionary Conserved Cysteines Function as cis-Acting Regulators of Arabidopsis PIN-FORMED 2 Distribution
Source: Int J Mol Sci. 2017 Oct 29;18(11):2274. doi: 10.3390/ijms18112274 (PMC5713244; doi:10.3390/ijms18112274)
Supplement: Supplementary file 1 [file ijms-18-02274-s001.zip › Retzer Supplementary Data/Supplementary Information.docx]

**Supplementary Information:**

**Supplementary Movie 1:** Animation of the PIN2 model, with the transmembrane domains in surface representation and the disordered loop as a ribbon, showing the location of the two cysteines, colored in pink. The movie was rendered with POV-Ray (Persistence of Vision™ Raytracer, Persistence of Vision Pty. Ltd. 2004;  [http://www.povray.org](http://www.povray.org/)).

**Supplementary File 1:** .pdb file, displaying the PIN2 model described in this manuscript
